# Functionality and acceptability of a wireless fetal heart rate monitoring device in term pregnant women in rural Southwestern Uganda

**DOI:** 10.1186/s12884-017-1361-1

**Published:** 2017-06-08

**Authors:** Godfrey R Mugyenyi, Esther C Atukunda, Joseph Ngonzi, Adeline Boatin, Blair J. Wylie, Jessica E. Haberer

**Affiliations:** 1 Mbarara University of Science and Technology, Mbarara, Uganda; 2 Mbarara Regional Referral Hospital, Mbarara, Uganda; 3 Massachusetts General Hospital, Boston, USA; 4 Harvard Medical School, Boston, USA

**Keywords:** Sense4Baby, Wireless fetal monitor, Electronic fetal monitoring

## Abstract

**Background:**

Over 3 million stillbirths occur annually in sub Saharan Africa; most occur intrapartum and are largely preventable. The standard of care for fetal heart rate (FHR) assessment in most sub-Saharan African settings is a Pinard Stethoscope, limiting observation to one person, at one point in time. We aimed to test the functionality and acceptability of a wireless FHR monitor that could allow for expanded monitoring capacity in rural Southwestern Uganda.

**Methods:**

In a mixed method prospective study, we enrolled 1) non-laboring healthy term pregnant women to wear the device for 30 min and 2) non-study clinicians to observe its use. The battery-powered prototype uses Doppler technology to measure fetal cardiotocographs (CTG), which are displayed via an android device and wirelessly transmit to cloud storage where they are accessible via a password protected website. Prototype functionality was assessed by the ability to obtain and transmit a 30-min CTG. Three obstetricians independently rated CTGs for readability and agreement between raters was calculated. All participants completed interviews on acceptability.

**Results:**

Fifty pregnant women and 7 clinicians were enrolled. 46 (92.0%) CTGs were successfully recorded and stored. Mean scores for readability were 4.71, 4.71 and 4.83 (out of 5) with high agreement (intra class correlation 0.84; 95% CI 0.74 to 0.91). All pregnant women reported liking or really liking the device, as well as high levels of comfort, flexibility and usefulness of the prototype; all would recommend it to others. Clinicians described the prototype as portable, flexible, easy-to-use and a time saver. Adequate education for clinicians and women also seemed to improve correct usage and minimise concerns on safety of the device.

**Conclusions:**

This prototype wireless FHR monitor functioned well in a low-resource setting and was found to be acceptable and useful to both pregnant women and clinicians. The device also seemed to have potential to improve the experience of the users compared with standard of care and expand monitoring capacity in settings where bulky, wired or traditional equipment are unreliable. Further research needs to investigate the potential impact and cost of such innovations to improve perinatal outcomes.

## Background

Worldwide, there are over 6.3 million perinatal deaths a year and of these, more than 4 million are stillbirth [1, 2]. One third of the stillbirths occur intrapartum and are largely due to birth asphyxia. Early neonatal deaths occur during the perinatal period, and typically have obstetric origins, similar to those leading to intrapartum stillbirths. Intrapartum stillbirths and early neonatal deaths from intrapartum events are rare in developed settings [3, 4]. Over 98% of these deaths take place in the developing world, with the risk of deaths in the neonatal period six times greater than in developed countries and over 8 times higher in the least developed countries [5–9].

In 2014, Uganda had a perinatal mortality rate of 40 per 1000 births [10]; 27% of these deaths resulted from birth asphyxia attributed to inadequate critical human resources, no action due to lack of reliable data, and/or delay in seeking help [11]. Though controversy remains over the benefit of continuous electronic fetal heart monitoring for most low risk laboring women, intermittent auscultation fetal monitoring is widely accepted as a critical element of intrapartum care [12].

**Fig. 1:**
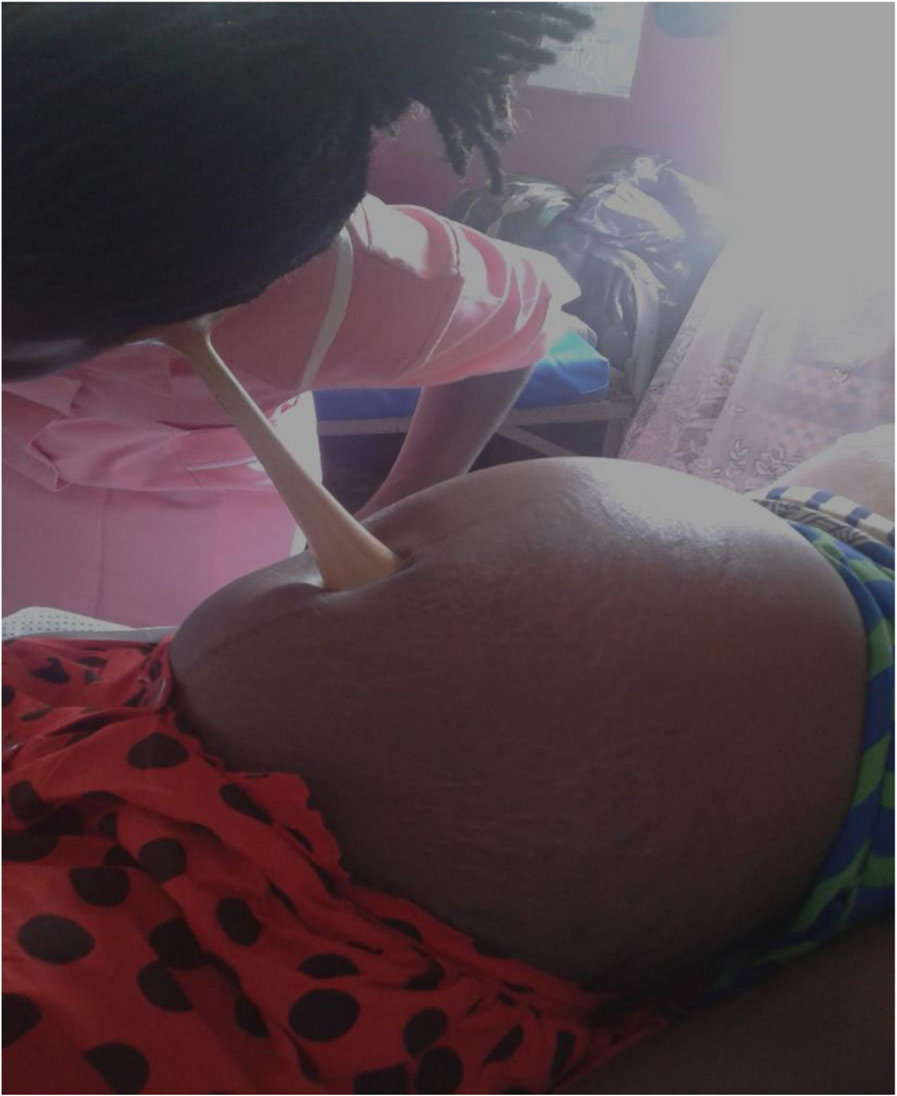
A Pinard’s stethoscope in use at Mbarara Regional Referral Hospital

In Uganda, the most common approach is a Pinard’s stethoscope (Fig. 1) used to manually listen to the pattern and frequency of fetal heart beat whenever deemed necessary. This tool has no visible corresponding tracings for review and fetal heart rate is estimated by pressing the tool firmly onto a mother’s abdomen, listening, and counting the heart beats. This method limits routine observations to one person at one point in time, is prone to errors, is restrictive to an examination couch since it requires the woman to be lying down, is time consuming and ultimately affects the continuity and frequency of observations amidst a shortage of human resource.

Innovative technology can improve efficiency of care amidst ongoing personnel shortages. Fixed and wired cardiotocograms (CTG) are available that provide visible and audible data in real time; however, the technology is immobile and has high maintenance costs [13], making it unsuitable and unavailable in low resource settings. With the improved availability of wireless signals in these settings, simple, user-friendly technology utilizing this wireless technology could overcome human resource challenges and improve monitoring, particularly if alerts are inbuilt to detect abnormal values. Consequently, this technology may have the potential to reduce the rates of perinatal deaths. Wireless fetal heart monitors that transmit fetal heart rate data to a central server remotely either through modems [14] or Bluetooth [15] have been developed to allow real-time assessment of labour. Pilot studies have found the Bluetooth technology to be acceptable and feasible to both women and clinicians in both outpatient and inpatient settings of developed countries [15, 16]. The recorded CTGs from a wireless fetal monitoring prototype technology were also found to be easy to read generally and the technology itself useful, likeable and recommended among pregnant women in an inpatient labour ward in USA. The wireless CTG monitoring further seemed to be friendly, with potential to improve outcomes.

This study set out to assess the functionality of a novel wireless prototype for measuring fetal heart rate and uterine contractions (cardiotocography) in a busy maternity unit in rural Southwestern Uganda. Acceptability was assessed from the perspectives of both the pregnant women being monitored and the clinicians interacting with the device.

## Methods

### Study design and setting

We conducted a cross-sectional observational study involving quantitative and qualitative methods of data collection at Mbarara Regional Referral Hospital, a publically-funded teaching hospital in rural south-western Uganda. We used a mixed methods study involving documentation of device functionality, as well as surveys to quantify acceptability and qualitative interviews to provide a more in depth understanding of factors related to acceptability [17]. The hospital employs 11 obstetricians and 22 midwives and performs over 10,000 deliveries annually [18] with approximately twenty beds located in each of two wings on the ward. Maternity departmental records of 2015 also indicate a maternal mortality rate of 270/100,000 live birth, caesarean section rate of 30% and a perinatal mortality rate of 56/1000. Although often incompletely implemented, all mothers in labour are ideally monitored using a partogram- a graph of labour parameters and cervical dilation over time with pre-printed alert and action lines designed to prompt intervention if a woman’s curve deviates from the expected course. The fetal heart rate is monitored manually per clinician judgment using the Pinard (Fig. 1). No electronic monitors are used on this ward, except for an ultrasound scan that is occasionally used to confirm presence or absence of regular cardiac pulsations, but not for monitoring labour. No wireless internet connection is available in the ward although cellular phone network coverage is available from multiple cellular phone companies. The ward walls are made of concrete (potential for interfering with wireless data transmission)

### Participants and recruitment

This study involved two types of participants: pregnant women and clinical staff observing the wireless fetal monitoring prototype. Using a convenience sample, we screened and recruited healthy non-laboring pregnant women, aged 18 and older, carrying a singleton gestation estimated to be at term and admitted to MRRH. The women were asked to wear the prototype for thirty minutes. Women who were unwilling to wear the monitoring device or those with a known infectious disease or hypersensitivity to materials found in the device were excluded. We also recruited a convenience sample of clinical staff to interact with the device and observe its use on recruited pregnant women. Informed written consent was obtained for all participants prior to enrolment. The study was conducted between May 2014 and August 2014.

### Wireless fetal monitor

**Fig. 2:**
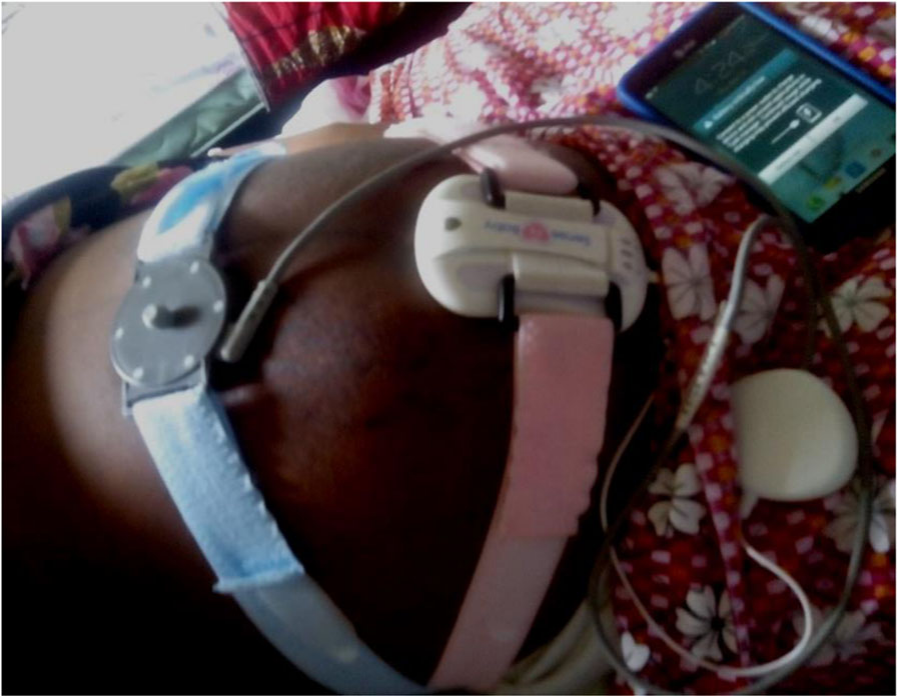
The wireless fetal heart monitoring prototype in use at Mbarara Regional Referral Hospital

The use of this wireless fetal monitor prototype has been previously described [15, 19] This device was developed by Gary and Mary West Health Institute, San Diego, California (Fig. 2). The device has received FDA approval for healthcare providers to use it to monitor expectant mothers and their fetuses during the antepartum period. FDA approval for use during active labor has not been approved. In our study we limited eligibility to non-laboring pregnant women. This non-invasive prototype technology uses Doppler-based technology to assess and record fetal heart rate combined with pressure sensors to track uterine contractions. Data is then transmitted via Bluetooth technology to a gateway device (either smartphone or tablet) where the cardiotocogragh is displayed and visualized in real time. In our study, we used the Samsung Galaxy Note (GT-N7000) as the gateway device. Once monitoring is complete, data is then transmitted from the gateway device to a secure web-based server for storage or further viewing through a web portal. Four steps are required to completely upload monitoring data on to the server; 1) save button, 2) a prompt to confirm completion of fetal heart rate recording session, 3) submit button to enable submission of data, 4) confirmation of data uploading on to the server for review via password access of any device that can access the web. Noteworthy, this study partly involves three of the authors in the Boatin study [15].

### Study procedures

All study procedures were performed by trained research midwives and resident obstetricians. At enrollment, study staff collected basic socio-demographic data and a brief obstetrical history from enrolled pregnant women using questionnaires and the medical record. The profession of the clinical staff (e.g. physician, resident, nurse, and midwife) was also noted.

Pregnant women were then asked to wear the prototype for 30 min. Study staff noted any technical challenges in obtaining and transmitting the fetal cardiotocographs. Following the monitoring session, a study obstetrician reviewed the cardiotocograph, prior to participant’s discharge from the study for safety, as well as quality. Clinical management decisions were made as per standard of care and no intervention was made on the basis of this technology prototype output. If there were concerns noted, they were confirmed by traditional methods (Pinard) used on the ward. The pregnant woman and clinical staff interacting with the device in use were asked to complete a brief questionnaire. Data from all questionnaires was transferred to a secure electronic database. The prototype tracings were not saved as part of the pregnant woman’s medical record. For this study, all women wore a pulse oximeter to allow differentiation from the fetal heart rate.

**Fig. 3:**
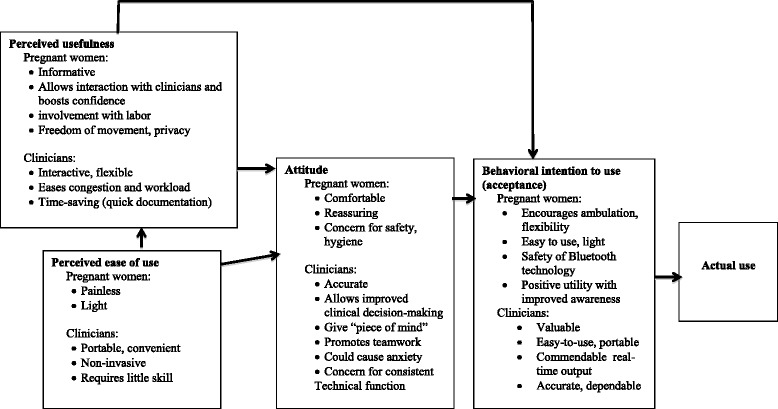
Technology acceptance model as applied to a wireless prototype cardiotocography technology in rural Uganda. The qualitative interviews of both pregnant women and clinician participants informed the model; actual use will be explored in future studies

Stratifying by age of the pregnant women (greater than or less than 30 years of age) to obtain a balance in participants, we identified five of the pregnant women participants for a qualitative interview. We used a convenience sample to identify seven of the clinical staff for qualitative interviews. These interviews further explored the perceptions, use, likes, dislikes, design and possible recommendations of this device, as guided by the technology acceptance model Fig. 3 [20]. This interview took place immediately after monitoring, lasted approximately 30 min in a private space, and was digitally recorded for transcription.

**Fig. 4:**
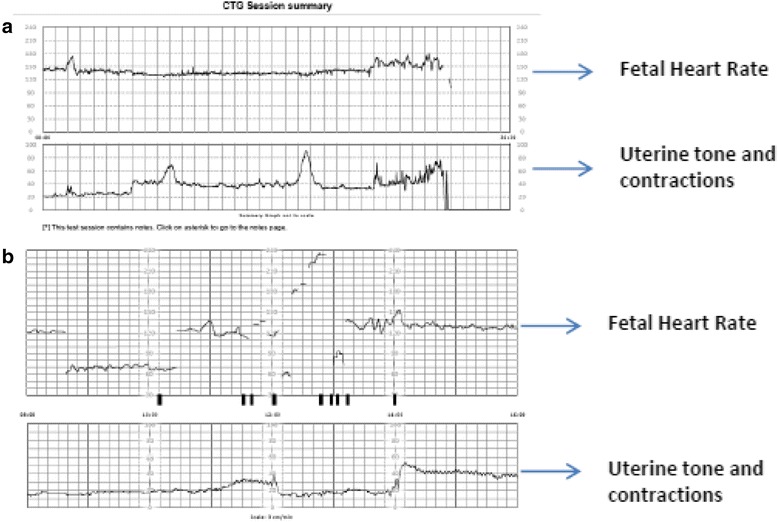
An example of **a** an easy-to-read and **b** a hard-to-read tracing

### Sample size

Our study was designed as a pilot to test functionality and feasibility. The number of participants was based on a convenience sample in which pregnant woman and clinicians were enrolled until sufficient data were obtained to assess the study goals and additional enrolment and data did not change study findings and interpretation.

### Data analysis

Quantitative data on participant characteristics, prototype acceptability and use were summarized and explored descriptively. Functionality was defined as the ability of the device to record a 30-min foetal cardiotocograph, upload this data to the cloud-based server, and retrieve and review the cardiotocograph. Three obstetricians rated the cardiotocographs for tracing quality and interpretability. Tracings were given a score from 1 to 5 (1 = difficult to read, 5 = easy to read) and the interclass correlation was calculated to assess agreement between raters.

### Qualitative analysis

Transcripts were derived from the qualitative interviews. Common cross-cutting categories were then generated from these transcripts and presented in line with the technology acceptance model.

## Results

### Participant characteristics

Fifty-two pregnant women were screened, of whom 50 were enrolled and 2 declined study participation. These women were mainly primigravid and aged 20–24 years with 22% having attained tertiary education. Other baseline and demographic characteristics are presented in Table 1. All seven clinicians screened were enrolled, of whom 6 were midwives and 1 doctor. They had been practicing for an average of 15.2 years.

**Table 1:** Baseline demographics and clinical characteristics of women enrolled for sense4baby device

Pregnant women		
Characteristic		n(%) or mean (±STD)
Age		
	18–19	6(2)
	20–24	25(50)
	25–29	17(34)
	30–34	2(4)
Parity		
	0	26(52)
	1	14(28)
	2	6(12)
	≥3	4(8)
Gestational age in weeks		40.1 ± 1.1
Marital Status		
	Married	45(90)
	Never Married	4 (8)
	Separated/Divorced	12(1)
Occupation		
	Unemployed	12(24)
	Self-employed/Unskilled	23(46)
	Professional	15(30)
Education		
	No Formal Education	2(4)
	Primary Education	17(34)
	Secondary Education	20(40)
	Tertiary Education	11(22)
Clinician Participants		
	Clinician age at enrollment	41 ± 11
	>18-34 yr	2(28.6)
	≥35	5(71.5)
Years in Clinical Practice at enrollment		15.2 ± 9.5
Clinical Position		
	Midwife	6(85.7)
	Doctor	1(14.3)
Time spent interacting with devices		
	1–15 min	0(0)
	16–30 min	7(100)

STD = standard deviation

### Functionality

All of the study staff were able to obtain the fetal heart rate and view the cardiotocographs transmitted through the Bluetooth technology on to the smart phone. Successful uploading of the monitoring data from the smart phone to the server, for storage occurred 100% of the time. The mean time from transmission to availability on the web portal was 2.2 min (±1.7). Delays in synchronizing data were mainly due to intermittent internet connectivity. Forty-six (92.0%) CTGs were successfully recorded and stored, while four (8%) of the CTGs were incomplete or unclear. Concrete walls seemed to have no effect on the wireless transmission of data.

### Tracing quality

Of the 46 cardiotocographs available for rating, the mean scores and standard deviation for readability were 4.71 ± 0.69, 4.71 ± 0.54 and 4.83 ± 0.38 (out of 5) for the three obstetrician raters respectively. Inter-rater agreement was high, intra class correlation 0.84 (95% CI 0.74 to 0.91). An example of an easy-to-read and hard-to-read CTG tracings is shown in Fig. 4.

### Acceptability

All women reported comfort and usefulness of the wireless technology prototype for fetal heart monitoring and contractions (Table 2). All women also liked the device and would recommend it to others for use. All clinicians found it likeable and very useful.

**Table 2:** Ease of use and acceptability of the prototype

Variable		Pregnant women (n(%))	Clinicians
Comfort wearing the device	Very comfortable	41(82)	N/A
	Comfortable	9(18)	N/A
	Neutral/ok	0(0)	N/A
	Somewhat bothersome	0(0)	N/A
	Very bothersome	0(0)	N/A
Usefulness of the device	Very Useful	40(80)	7(100)
	Useful	10(20)	0(0)
	Somewhat useful	0(0)	0(0)
	Not at all useful	0 (0)	0(0)
Acceptability of device	I really like it	45(90)	4(57)
	I like it	5(10)	3(43)
	Neutral/OK	0(0)	0(0)
	I do not like it	0 (0)	0(0)
	I really do not like it	0 (0)	0(0)
Recommending use to others	I definitely would	49 (98)	6(86)
	I wouldn’t care one way or the other	1(2)	1(14)
	I definitely would not	0 (0)	0(0)

### Perceptions about the wireless technology prototype

The opinions of both pregnant women participants and clinicians are presented below according to the Technology Acceptance Model (Fig. 3).

### Perceived usefulness

#### Pregnant women participants

All women liked the technology because it helped them to interact with their health care providers and know more about their health and that of their babies. This interaction seemed to facilitate women’s involvement in management of their labour as well as boost their confidence in observing and knowing that their babies were okay and obtain help in case anything visibly wrong was displayed.

“I loved the device a lot because I got to interact with the midwives and doctors as they talked to me about my health and my baby’s condition all the time, something that has never happened to me before. In fact, I was very confident as I was able to see my baby’s heart beat right there on the screen and this meant a lot to me as a mother because I knew my baby was okay. It’s like I got to participate in the safe delivery of my baby which means a lot to me … I was also confident to know I could be helped if something wrong visibly happened.”

Additionally, the device also was thought to ‘constantly’ inform their care takers of their babies’ health status, facilitating a prompt action to avoid complications in case it happened unlike the Pinard stethoscope which was thought to be non-informative and depended only on individual judgment from the clinician using it. This constant information reportedly kept women relaxed and made them feel good being a part of the process to deliver their babies safely.

“Unlike the other one [Pinard] which is placed on your abdomen and the midwife just tells you that your baby is fine and that is all without hearing anything yourself, this device makes me feel good and relaxed as my care takers and I are continuously able to see and discuss how my baby is doing and take action.”

#### Clinician participants

The technology was also described by clinician participants as interactive and able to facilitate or improve team work between staff and patients. They stated that the prototype allowed a lot of the needed flexibility and mobility amongst their patients while posting the needed data in real time.

“This (technology) is very interactive. It’s very useful than any other devices I have used in my entire practice. Information is continuously displayed for you to see, review and promptly share it with all the attending staff or the mother in real time … In fact, its flexibility allows pregnant mothers to move about their businesses during monitoring unlike the traditional old fashioned stethoscope that needs one to keep still, silent, physically be present on an examination couch.”

The wireless nature of the device was further seen to greatly ease congestion and workload that could in turn improve labour monitoring and improve health outcomes generally amidst the limited human resource at the hospital. Clinicians also noted the ability of the wireless device to facilitate their attendance to other routine work or more mothers simultaneously, which to them could potentially save time and lives in the long run. The ability of the technology to provide fast and quick documentation of fetal well-being in real time was seen to be highly valuable in facilitating quick informed decisions needed for improved health outcomes. These clinician participants further and specifically likened the device’s ability to give a permanent record in real time to be a great accessory in medical-legal cases related to maternal/fetal deaths and or complications.

“It really saves a lot of time, which can be used to interact or attend to other mothers in labour or other emergencies as you monitor them closely and simultaneously amidst our limited human resource … we could save time, save more lives and definitely reduce my workload and stress. In fact, it makes my work easier, enjoyable and manageable.”

“This device is very useful and valuable in recording data continuously and permanently in real time. This helps you to make quick and informed decisions immediately to avoid many fresh still births, compared to the stethoscope which estimates individual rates at a time manually and plotting these rates manually onto a partogragh in a patient’s file, which can be very tiresome and problematic when one forgets to do or record observations timely in such a place with few personnel … this could also have some protection in case of a court case involving a perinatal deaths.”

### Perceived ease of use

#### Pregnant women participants

Labouring women also liked the technology and thought it was painless and light unlike the Pinard stethoscope, which they described as uncomfortable and painful. These perception soften encouraged them to use this technology through the monitoring period.

“The midwife always maneuvers physically on my abdomen to listen to the fetal heart rate several times which causes me a lot of pain and discomfort. With this [device], there was no pain … I also liked it because it is not heavy on me so I can easily use it as long as necessary.”

A sense of freedom of movement and flexibility offered by the wireless device during labour was also greatly appreciated by all women compared to the traditional method which they described as heavy and restrictive. These mothers also felt the device boosted their privacy, something that helped them to have a positive mind during labour.

“this [device] is very light, allowing me to move around freely as I am being monitored and not always stuck here [on my bed] physically unlike the other one [Pinard] … I did not evenhave to undress here on the general ward to be examined all the time which makes me feel uncomfortable and sometimes embarrassed. Instead, I was able to relax, move around as I waited for the delivery of my baby.”

#### Clinician participants

All clinician participants described the device as portable, convenient and non-invasive. They also felt it was easy-to-use and with no need for specialized skill or training to successfully install and use. Its non-invasive and easy-to-use nature was further said to be appealing to clinicians. According to a senior midwife,

“I like the fact that it is simple to learn by everyone, light, portable, convenient and easy to use on someone and it does not take a lot of time or skill to learn or set up. The fact that it is a non-invasive device also makes it very appealing and easy to use.”

### Attitude

#### Pregnant women participants

According to the women, the device was thought to be well designed, comfortable and ‘very re-assuring’ since it indicated the fetal heart rate on the screen in real time for them to see. This to them was very fulfilling.

“I like the design because it is tied comfortably around my abdomen, so close to the baby so it gives very close and accurate information about my baby’s condition inside for everyone to see. This for me is very fulfilling and re-assuring. I like it a lot.”

Women reported that the device’s wireless nature andinteractive attributes reduced anxiety about the ‘unknown’. Their sense of feeling that ‘everything was always visible and under control’ seemed to be an additional reassurance for many women.

“My anxiety reduced while I used this device since I did not have to worry about anything bad happening to me or my baby unknowingly…For the first time, I could see my babies’ heart beat all the time and interact with midwives and doctors whenever I wanted. This really gave me the reassurance and courage to be strong and go about doing my other things knowing my baby was okay and everything was visible and under control.”

A few women however expressed some concerns about safety and hygiene of the device. According to one mother for example,

“I know some women may be worried about possible radiations in the device…they could also be worried about some belts being worn by many women without adequate cleaning.”

### Clinician participants

Clinicians thought the technology prototype was accurate and dependable in making objective and correct clinical conclusions in real time that in turn could ease workload and improve health outcomes in the long run. The device’s consistency and ability to detect and record any abnormal variations in fetal heart rate in real time was seen to encourage quick informed decisions to avert complications and or deaths. According to a senior obstetrician,

“The readings are accurate and dependable. It gives us results in real time throughout the monitoring period, inclusive of abnormal variations in fetal heart rates which obviously helps us to make prompt informed decisions. This is very key in successful management of labour, besides, the added time and workload involved in investigating false alarms or late complications are generally minimized.”

Some clinician participants thought that the device’s flexibility, non-invasive and wireless nature gave women a sense of comfort and a peace of mind that could facilitate good health outcomes.

“I don’t have to call up mothers all the time for examination. Since its non-invasive wireless technology, she can remain relaxed in her bed as she is being monitored and this comfort in its use gives mothers a peace of mind while going through labour which is good.”

Other clinicians observed that the wireless device promoted continuous information sharing, discussion, improved team work and this was seen to have the ability to greatly improve the work experience amongst the staff and laboring women.

“We are few midwives here and we struggle a lot using the old fashioned Pinard to monitor all these mothers in labour. I am sure if all midwives had access to this device with continuous information sharing and discussion, I know it would lessen our work load, promote team work amongst us and inform our timely decisions…Every woman that comes here could go away healthy, with a healthy baby, something which is absolutely fulfilling as a midwife.”

Other health care providers thought the device could be liable to inconsistencies, sometimes providing poor or no readings at all especially when confronted with dead/uncharged batteries and internet connectivity problems. A *senior house officer* noted,

“I think the device will give me correct and accurate data only if the phone is well charged and the device is well connected. This means always charging the phone and having data on the internet to be able to work effectively.”

### Behavioral intention to use the device

#### Pregnant women participants

The pregnant women participants expressed an interest in using the device with proper explanation of the technology. The device’s perceived ability to always ‘tell what is happening with the unborn baby’ seemed to positively influence its utility. Its use of Bluetooth technology, which was known to many women, also seemed to add a sense of safety among the users.

“If the nurse explains to them [women] clearly how the device works and its use, I am sure many of us will like to use it a lot because it works for our own good by always telling you what is happening with our babies…besides, it uses Bluetooth just like my phone here and so I think it’s safe.”

The device’s perceived ease of use through its ability to encourage ambulation of pregnant women through its wireless connection seemed to greatly encourage its good uptake and use among the pregnant women.

“It did not have fixed wires attached to me all over the place, neither was I stuck to one place. I was able to freely move around during my labour without any problems. I think this would make it easy for me to use it always”*.*

#### Clinician participants

All clinicians combined perceptions of ease of use and usefulness to describe the device’s high utility value and their intention to use the prototype. According to a senior obstetrician for example,

“It’s very simple, portable and generally easy-to-use… It’s definitely commendable and looks like one I can use any day any time as it makes my work easier and bearable…every doctor or midwife would definitely appreciate its output and always use it if it was available.”

However, a few clinicians felt that some mothers could be bothered about the safety of the device and its effects to their unborn babies if not sensitized well, which could in turn increase anxiety.

“Some of the mothers may be anxious about the safety of these new electronic gadgets…but I think many will definitely like it and accept to wear it when well sensitized about its [device] safety since it also uses Bluetooth [technology] that is known to many people here”

Additionally, some clinicians expressed concerns about the device’s inability to allow storage at the gateway stage, limiting them to review the tracings displayed on the smart phone continuously only within the recommended distance from the Bluetooth-enabled technology as opposed to reviews online only after data is successfully secured on the server.

“Working on this our big ward with very few staff is difficult. I may be attending to mothers on the other side where the Bluetooth on my phone doesn’t pick any signals from the device directly unless recorded data is saved online first…that way, I may not be able to see any tracings in real time wherever I am and act accordingly in case of a serious problem”*.*

## Discussion

We demonstrated the functionality and acceptability of a novel wireless monitoring device for measuring foetal heart rate and uterine contractions amongst pregnant women and health care workers at a publically-funded teaching hospital in rural south-western Uganda. We found that over 90% of CTGs were successfully recorded and stored within two minutes. Importantly, we found high readability and high inter-individual agreement on the quality of the tracings. We observed that all women liked the prototype and described it as comfortable, flexible and useful and would therefore recommend it to others. We also observed corresponding reports from clinicians who described the prototype as portable, flexible, easy-to-use, fast, accurate and dependable. They felt it presents an important opportunity to save time, facilitate prompt informed decisions and ease their work load in such a resource constrained setting. However, prototype safety, battery issues and regular internet connectivity to enable constant availability of tracings were noted by both clinicians and women as concerns if the technology were rolled out.

Like a prior study done in a USA inpatient obstetric setting [15] we found high acceptability, desirability and functionality of the prototype in both the pregnant women and clinicians. Additionally, all women in this prior study also seemed to like the great comfort, privacy and mobility that the wireless fetal heart monitor offered them during labour, suggesting promising desirability and uptake. However, there were major differences. Whereas clinicians and pregnant women’s experiences compared immobile/wired CTGs and wireless technology prototype in a USA setting [15], our participants’ experiences compared the prototype with the routinely used Pinard stethoscope. Our study was also conducted among relatively healthy full-term pregnant women carrying singleton gestations in latent labour admitted to a rural resource limited maternity ward in south western Uganda. Whereas the USA study noted design (for example; “*small screen for older eyes*”, small size) as one of the major concerns for women users, all participants in our study seemed to like the design, with major concerns noted as safety, battery issues and intermittent internet connectivity problems that would affect timely and continuous transmission and display of data. Connectivity problems with this network-enabled technology have previously been reported but were remedied by enabling the queued data to appropriately be uploaded once a good signal was available [15]. In our study, delays in synchronizing data happened once a week on average and were overcome using pre-paid airtime on multiple network SIM cards inserted in a study smart phone to buy and secure data bundles, which minimized delays mainly because of its current network consistence in connectivity locally. Use of the SIM cards avoided interference reported by our US sister study, which used a general hospital Wi-Fi system [15].

Noteworthy from this study, was the perceived interactive nature of the technology that facilitated continuous discussions of the participants’ and their babies’ wellbeing with their clinicians, a practice that is generally uncommon in Uganda. This ongoing involvement in the management of their labor, facilitated by the displayed tracings from this technology seemed to have created a sense of satisfaction and feelings of ‘constant awareness’ from its use compared to a routine sense of ‘fear of the unknowns’ reportedly emanating from traditional methods. This overall perceived usefulness of the prototype therefore seemed to keep the laboring women confident, relaxed and positive minded and could therefore pose a great opportunity for its high uptake. However, as expected from introduction of any new technologies, safety concerns were noted by clinicians and pregnant women, which seemed to be diminished through careful sensitization and training prior to use.

The technology used in the prototype functioned well in a low-resourced setting. The Bluetooth technology, which is widely available, seemed to minimize perceived safety concerns from pregnant women and clinicians. The transmission time was consistently short with reliable connectivity using two cellular networks in the study phone interchangeably. One problem, however, was the fact that the prototype interface did not allow for storage at the gateway stage for clinicians to review. In a non-research busy labour ward, a clinician may have difficulty staying near the device to observe the CTGs displayed on the smart phone in real time. Boatin and colleagues recommended real time transmission of the CTG from the gateway to a central monitor as a potential means for resolving this problem, which could also potentially work in this setting [15].

Our study has important strengths. First, it documents novel perspectives of pregnant women and clinicians on the acceptability and functionality of this prototype technology in a rural low- resourced setting, which has not been studied before. Second, the mixed methods design (including quantitative and qualitative aspects) provides a rich and deep understanding of the issues involved with technology uptake.

We believe that the usefulness of a device depends on both the perspectives of the care providers and the patients. Indeed, no device will be used in practice if patients object to it. Moreover, other studies have taken similar approaches to studying technology acceptance [21, 22]. Several limitations were also observed in our study. First, the small sample size could affect the generalisability of the findings, although we found consistency in both the quantitative and qualitative findings. Whereas this study demonstrates potential for the wireless foetal monitoring to be greatly acceptable in an inpatient low resource setting, we only assessed a 30-min period per pregnant woman. Additional study with integration of the technology into day-to-day work flows is needed. Importantly, we also did not assess clinical benefit in preventing or mitigating poor foetal outcomes, which should be explored now that the functionality and acceptability of this prototype technology have been demonstrated.

## Conclusions

This wireless prototype cardiotocograph functioned well in a low-resourced setting and was found to be acceptable and useful to both pregnant women and clinicians. The device also had potential to improve the experience of the users and expand monitoring capacity compared with standard of care. This study is the first to our knowledge that demonstrates the use of a wireless cardiotocograph in a low resource setting. It is also the first study to query acceptability of such a device among patients and clinicians in a low resource context. Our study demonstrated that this novel wireless prototype cardiotocograph functioned well and was acceptable and useful to both pregnant women and clinicians, with potential of improving the experience of the users. This device will potentially expand monitoring capacity compared with standard of care especially in resource constrained environment. Further research needs to investigate the potential impact and cost of such innovations to improve perinatal outcomes.

## Abbreviations

FHR: Fetal hart rate
CTG: Cardiotocography
